# The Influence of the Lactation Period and the Type of Milk on the Content of Amino Acids and Minerals in Human Milk and Infant Formulas

**DOI:** 10.3390/foods12193674

**Published:** 2023-10-06

**Authors:** Aleksandra Purkiewicz, Małgorzata Stasiewicz, Jacek J. Nowakowski, Renata Pietrzak-Fiećko

**Affiliations:** 1 Department of Commodity Science and Food Analysis, Faculty of Food Science, University of Warmia and Mazury in Olsztyn, Plac Cieszyński 1, 10-726 Olsztyn, Poland; renap@uwm.edu.pl; 2 Department of Animal Nutrition and Feed Management, Faculty of Animal Bioengineering, University of Warmia and Mazury in Olsztyn, Oczapowskiego 5, 10-719 Olsztyn, Poland; malgorzata.stasiewicz@uwm.edu.pl; 3 Department of Ecology and Environmental Protection, Faculty of Biology and Biotechnology, University of Warmia and Mazury in Olsztyn, Plac Łódzki 3, 10-727 Olsztyn, Poland; jacek.nowakowski@uwm.edu.pl

**Keywords:** breast milk, amino acid, minerals, lactation period, breastfeeding

## Abstract

(1) Background: This study investigated the effect of the lactation period and the type of infant formula on the content of amino acids and selected minerals in an infant’s food; (2) Methods: The study material consisted of breast milk (colostrum, *n* = 38; transitional milk, mature milk, *n* = 38) and three types of infant formulas (for first and follow-on feeding). Amino acid content was determined using an automatic amino acid analyzer, while minerals were determined by the atomic absorption spectrometry (AAS) technique; (3) Results: Breast milk and infant formulas contained a full range of essential amino acids. In most cases, the content of individual amino acids and minerals decreased with increasing lactation. In infant formulas, there were higher contents of phenylalanine, glutamic acid, proline, serine, and tyrosine in follow-on milk (*p* < 0.05). The EAA/TAA ratio in breast milk and infant formulas was similar, but the milk differed in their qualitative composition. Infant formulas contained levels of individual minerals that were several times higher—especially Mg, Ca, Mn, and Fe.; (4) Conclusions: Colostrum is more concentrated, and the level of amino acids and minerals is higher in it; as the milk matures, it decreases. In most cases, the content of individual amino acids and minerals is higher in infant formulas than in human milk, which is established through strict Codex Alimentarius procedures to ensure the proper development of infants.

## 1. Introduction

Nutrition plays an important role during infancy because of the high intensity of growth and the rate of physiological development. It is reported that early nutrition has a programming effect on health in adult life due to the presence of bioactive ingredients such as proteins, fatty acids, vitamins, minerals, growth factors, and antioxidants [1]. Breast milk is recommended for infant feeding because of its unique composition, which adapts individually to the infant’s needs. It is recommended that infants be exclusively fed breast milk until at least 6 months of age [2]

Protein is an important component of human milk and determines its nutritional value. It is reported that mother’s milk is a source of nearly 300 proteins with unique functional properties. They are a source of amino acids, which have properties that ensure the rapid growth of an infant. In addition to their building function, they are responsible for facilitating the digestion of food by increasing the absorption of other nutrients and protecting the child’s body against pathogens, bacteria, viruses, and yeasts [3]. The quality of a protein depends on its amino acid composition. Amino acids are divided into essential and non-essential categories [4]. Infant formulas contain a higher protein content compared to breast milk, in accordance with established legislation. The amino acid content contained in infant formulas must be at least equal to the amount found in human milk [5]. Furthermore, the protein profile of human milk varies depending on the lactation period. The highest protein content is found in colostrum (1.4–1.6 g/100 mL), whereas it decreases in transitional milk (0.8–1.0 g/100 mL) and mature milk (0.7–0.8 g/100 mL) [3]. The tendency for the composition of milk to change in relation to the lactation period is physiologically determined. Protein content decreases in favor of fat, which causes an increase in the energy content of breast milk.

Breast milk contains these macro and microminerals that are crucial for the newborn’s development: sodium, potassium, magnesium, calcium, chlorine, zinc, copper, manganese, selenium, and iodine [6]. Despite the low ash content of breast milk, the minerals found are highly bioavailable [7]. Similar to amino acids, the mineral content of human milk is not constant and depends on the lactation period. The levels of selected minerals, especially iron, sodium, selenium, and zinc, decline during breastfeeding [6].

Infant formulas are recognized as a suitable alternative to human milk by the World Health Organization (WHO), the American Academy of Pediatrics (AAP), and UNICEF when breastfeeding is not possible [2]. Depending on the age of the infant, there is a classification of infant formulas for first (up to 6 months of age) and follow-on feeding (from 6 months of age) [8]. The composition of infant formulas is still being improved to replicate human milk as closely as possible, according to the content of the unique ingredients in breast milk. The amino acid composition of infant formulas should be composed in such a way that the content of essential amino acids is at least equal to their content in human milk [8]. On the other hand, the mineral content of infant formulas needs to be continuously adjusted to the age of the child by changing the manufacturing technology [7].

There are many published data on the content of amino acids and minerals in infant food. Previous studies have established that amino acids and minerals fulfill an important role in newborn development, and their content is determined by the stage of lactation [9]. However, data about the comparison of amino acids and minerals in human milk and infant formulas in terms of the lactation period are limited. This research had the following aims: (1) to determine the level of protein, amino acids, and selected macro and microminerals in breast milk and infant formulas from different manufacturers; (2) to assess the influence of the lactation period and the type of infant formula on the content of these compounds; (3) to compare the content of amino acids and selected minerals in infant foods.

## 2. Materials and Methods

### 2.1. Chemicals

Sulfuric acid (VI), sodium hydroxide, boric acid, formic acid, hydrochloric acid, and hydrogen peroxide were obtained from Sigma Chemical Co. (St. Louis, MO, USA). Amino acid standard solution AASS-18, amino acid standards for protein hydrolysate A 2908, L- methionine sulfone, L-cysteic acid, and L- norleucine were purchased from Sigma Aldrich (St. Louis, MO, USA). Nitric acid and heptahydrate lanthanum chloride were obtained from Merck (Darmstadt, Germany).

### 2.2. Milk Sample Collection

The research material consisted of samples of breast milk and infant formulas from three manufacturers. Breast milk samples were collected from 100 local women currently living in the East-northeast region of Poland. The inclusion criteria for this study were the good health of the women, the absence of contraindications to breastfeeding, and easy delivery. The women interviewed were asked to fill out a questionnaire about personal data (first and last name, age, place of residence), body weight and height, current illnesses, medications frequently taken, and information about pregnancy and breastfeeding (the type of birth, date of the birth of the child, stage of lactation, date of milk withdrawal, sex of the child, weight and height of the child, and number of children born). All participants provided written informed consent regarding their participation in this study.

Milk samples (50 to 100 mL) from each woman were collected in sterile glass bottles, using electronic breast pumps immediately after breastfeeding. The women were instructed to store the milk in a tightly closed container in the refrigerator (4 ± 1 °C) and refill it during the subsequent breastfeeding session if it was not possible to collect the appropriate amount of milk during one time. The milk samples were stored at −30 °C in glass-sealed, sterile containers until chemical analysis. The collected material included 43 human milk samples from the first lactation period (colostrum—breast milk until a few days after birth) and 57 human milk samples from the second and third lactation periods (transitional milk/mature milk—breast milk until 2 weeks after birth/from 4 weeks of breastfeeding) were collected. Due to the insufficient number of samples for analysis, analytical tests were carried out on 76 samples of human milk (38 from the 1st lactation period and 38 from the 2nd and 3rd lactation periods). Additionally, three producers of infant formulas for infants’ first and follow-on feedings were included in the research materials. Infant formulas were obtained from the Olsztyn market. Each of the infant formulas for first and follow-on feedings were based on cow’s milk and came in the form of powders that were reconstituted with water according to the instructions on the package. Table 1 summarizes the ranges of the nutritional values of the infant formulas analyzed for first and follow-on feedings and the method of their preparation. Information in this table was obtained from the original packaging of the products. In this conducted study, a conventional infant formula with a low degree of protein hydrolysis was used.

### 2.3. The Determination of Protein Content

The human milk samples were removed from the freezer, thawed, and mixed rapidly. The tested breast milk samples were heated to 40 ± 1 °C. Each infant formula was prepared for this study according to the instructions on the package. Protein was determined by the Kjeldahl method [10]. The nitrogen content in the samples (%N) was converted to total protein content using a conversion factor of 6.25.

### 2.4. Amino Acid Profile

Amino acids were determined using a Biochrom 20 Plus automatic amino acid analyzer (Cambridge, UK) [11], according to the method described by Landi et al. [12] and European Commission Directive 98/64/EC [13]. The hydrolysis of the proteins of the test samples was carried out in glass ampoules under a nitrogen atmosphere in 6 mol HCl at 110 °C for 24 h. An amino acid standard solution AASS-18, amino acid standards for protein hydrolysate A 2908, L-methionine sulfone, L-cysteic acid, and L-norleucine were used as amino acid standards. Sulfur amino acids (cysteine and methionine) were determined in the form of cysteic acid and methionine sulfone after the oxidation of the samples with peracid (a mixture of peracid HCOOH and perhydrol H_2_O_2_). The formic acid was then decomposed and evaporated, and the samples were hydrolyzed in 6 M hydrochloric acid at 110 °C for 24 h under a nitrogen atmosphere. Tryptophan was determined according to Polish standards [14].

### 2.5. Mineral Content

The mineralization of the samples was carried out according to the method described by Whiteside and Miner [15]. Standardized milk samples (10 mL) were weighed to quartz crucibles and dried in a laboratory dryer (Memmert UF30, Schwabach, Germany) for 8–12 h at a temperature of 60 °C. The samples were then carbonized on an electric cooker for 8 h in 480 °C and then placed in a muffle furnace (Nabertherm GmbH, Lilienthal, Germany) to obtain a white-gray ash (with the temperature rising gradually from 100 °C to 500 °C).

The ashes were heat dissociated in 1M HNO_3_ (Merck, Darmstadt, Germany), transferred to 25 mL volumetric flasks, and filled with deionized water. Reagent samples were prepared in parallel to the test samples. The mineral content was determined in the mineralizations obtained (directly or after their dilution).

The contents of copper (Cu), manganese (Mn), iron (Fe), zinc (Zn), calcium (Ca), and magnesium (Mg) were determined by the flame atomic absorption spectroscopy (AAS) method (acetylene–air flame). Analyses were conducted using an atomic absorption spectrometer iCE 3000 SERIES-THERMO (Thermo-Scientific, Hemel Hempstead, Hertfordshire, United Kingdom) equipped with a GLITE data station, background correction (deuterium lamp), and suitable cathode-ray tubes. To determine the Ca content, the addition of a 10% heptahydrate lanthanum chloride LaCl_3_*7H_2_O (Merck, Darmstadt, Germany) in an amount providing a final concentration of La^+3^ of 1% was used.

Sodium and potassium (Na and K) contents were determined by the emission technique (acetylene/air flame). Analyses were conducted using an atomic absorption spectrometer iCE 3000 SERIES-THERMO (Thermo-Scientific, Hemel Hempstead, Hertfordshire, UK) equipped with a GLITE data station and operating in the emission system.

### 2.6. Statistical Analysis

Values were expressed as the mean ± standard deviation (SD). The distributions of the studied variables in the samples were tested against a normal distribution using the Shapiro-Wilk test, and the homogeneity of variance was tested using the Levene’s test or the Bartlett test. Comparison of the collected quantitative data between the distinguished groups was performed using non-parametric Kruskal–Wallis and Dunn’s post hoc tests, and in the situation of comparing two samples, depending on the fulfillment of assumptions, a Mann–Whitney test or parametric Student-t-test, Cochran-Cox tests were used. The level of significance was assumed at *p* < 0.05. Statistical analysis was performed using Statistica 13.1 (Statsoft Inc., Tulsa, OH, USA).

## 3. Results and Discussion

### 3.1. Protein Content

**Figure 1:**
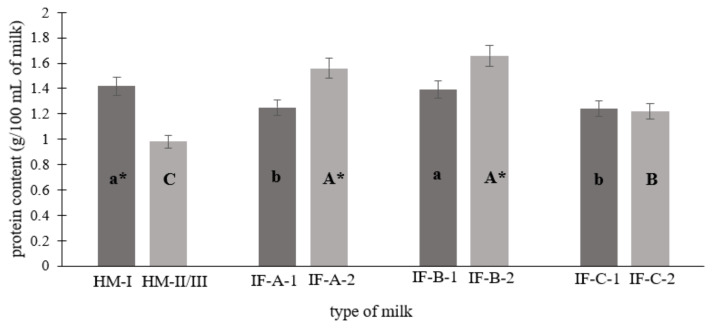
The protein content (g/100 mL of milk) of the human milk and infant formulas studied. Abbreviation: HM-I—human milk (^I^ stage of lactation), HM-II/III—human milk (II and III stage of lactation); IF-A-1/IF-B-1/IF-C-1—infant formulas from different manufacturers for the first feeding; IF-A-2/IF-B-2/IF-C-2—infant formulas from different manufacturers for the follow-on feeding. Means with different letters (a,b,c/A,B,C) are significantly different (*p* < 0.05). Means a,b,c specify differences between HM-I, IF-A-1, IF-B-1, and IF-C-1; means A,B,C specify differences between HM-II/III, IF-A-2, IF-B-2, and IF-C-2. Values marked with * indicate differences in protein content between breast milk from the 1st and 2nd/3rd lactation period or between the first and follow-on infant formulas.

Figure 1 shows the total protein content in human milk (1st, 2nd, and 3rd stages of lactation) and infant formulas from different producers (for the first (1) and follow-on (2) feeding). Breast milk from the 1st stage of lactation was characterized by the highest protein content, which was significantly greater than the IF-A and IF-C first-feeding (1) infant formulas (*p* < 0.05). The opposite tendency was observed in human milk from the 2nd and 3rd stages of lactation, where the level of protein was the lowest compared to IF-A, IF-B, and IF-C follow-on (2) feeding infant formulas (*p* < 0.05).

There was an over 30% decrease in protein content in transitional and mature milk compared to colostrum (*p* < 0.05). As lactation progresses, the composition of breast milk changes. Mature breastmilk holds more water and is less concentrated than colostrum. Gradually, the content of lactose and fat in it increases, and the content of protein decreases. In addition, it is an excellent source of vitamins, minerals, immunoglobulins, leukocytes, and growth factors. Studies by Lönnerdal et al. [9] indicate that the protein concentration in colostrum is at a level of approximately 1.4–1.6 g/100 mL, decreases to approximately 0.8–1.0 g/100 mL at 3 to 4 months of breastfeeding, and then decreases to 0.7–0.8 g/100 mL at 6 months (mature milk). The results of the meta-analysis and systematic reviews about breast nutrient composition are compatible with the research conducted. Both sources indicate higher protein content in colostrum than in transitional and mature milk [16,17].

The protein content in the infant formulas ranged from 1.22 to 1.66 g/100 mL. These contents were similar to the protein content in the infant formula determined by Trabulsi et al. [18]. Moreover, the determined protein content met the requirements of the Codex Alimentarius and the EU Commission [8,19]. There was a significantly higher protein content in follow-on feeding infant formula compared to second- and third-stage breast milk (*p* < 0.05). It is proven that human milk has a substantially lower concentration of protein than the milk of other mammals. On the other hand, human milk provides a unique source of essential amino acids, which allows infants to meet their full protein needs [20]. The quality of the protein and its composition are especially important during infancy. It is stated that during infancy, essential amino acids comprise one-fifth of the protein requirements. [20] For this reason, the higher protein content in follow-on infant formulas may be related to the growing needs of the infant and the higher demand for amino acids, especially essential ones. The greater addition of protein that is added to infant formulas is needed to ensure that the infant’s demands for all amino acids are met [18].

### 3.2. Essential Amino Acid Profile

Results from the study conducted confirm that the lactation period has a significant impact on the variation in the amino acid composition of human milk. The results of the essential amino acid concentrations in human milk and infant formulas are shown in Table 2.

Breast milk contained all nine essential amino acids. Human milk had the highest amounts of leucine and the lowest amounts of tryptophan and methionine, as in the studies of Zhang et al. [17]. In the case of human milk, there was a decrease in the content of selected essential amino acids in transitional and mature milk compared to colostrum. These differences were significant for tryptophan, leucine, isoleucine, phenylalanine, threonine, valine, and methionine (*p* < 0.05). The reduction in the level of amino acids in mature milk is a natural physiological reaction and is correlated with the changing protein needs of the developing infant. It is reported that the greatest decline in the concentration of amino acids is observed during the first four months of lactation [9]. In mature milk, amino acid content stabilizes and is maintained in such amounts. Previous studies also show lower levels of amino acids in mature milk compared to colostrum [17,21]. In some cases, the opposite trend was noted: the level of amino acids was higher in mature milk than in colostrum, which can be explained by the occurrence of possible temporary changes in the amino acid composition of breast milk [21]. Among essential amino acids, the most significant differences in content were noted in the case of lysine. In each infant formula, both first (1) and follow-on (2), the concentration of lysine was significantly higher compared to breast milk (type I: Dunn test: A vs. HM—z = 5.979, *p* < 0.00001; B vs. HM—z = 4.425, *p* = 0.00006, C vs. HM—z = 4.273, *p* = 0.0001, type II: Dunn test: A vs. HM—z = 8.989, *p* < 0.00001, B vs. HM—z = 6.043, *p* < 0.00001, C vs. HM—z = 5.479, *p* < 0.00001). It should be emphasized that the levels of some essential amino acids, especially tryptophan, methionine, histidine, and phenylalanine, show changes in the circadian cycle. In the case of tryptophan, which plays an important role as a sleep promoter and improves the quality of the baby’s sleep [22,23], its highest amounts in breast milk are at 3–4 a.m [24]. Therefore, the concentration of tryptophan depends on the time of feeding [22].

According to the Codex Alimentarius, infant formulas—including first and follow-on—are produced according to strictly defined standards [8,19]. The dominant amino acids in infant formulas were leucine and lysine, while tryptophan was present in the smallest amount. Despite the same trend in the content of leucine and tryptophan in human milk, infant formulas contained a significantly higher content of lysine (*p* < 0.05). In addition, higher levels of histidine, leucine, threonine, valine, and methionine (*p* < 0.05) as well as tryptophan (*p* > 0.05) were noted. The recommended content of essential amino acids in infant formulas should at least be equal to those contained in human milk protein. Commonly available infant formulas are made from cow’s milk, whose amino acid composition differs from human milk. For this reason, infant formulas have a higher total protein content to protect the infant from essential amino acid deficiencies [8,19].

The content of essential amino acids in infant formulas varied depending on the manufacturer. Infant formula IF-A, both first (1) and follow-on (2) feedings, contained the highest amounts of histidine, tryptophan, leucine, phenylalanine, threonine, lysine, valine, and methionine. The content of histidine, tryptophan, and leucine in infant formula IF-A for first feeding (1) and the content of histidine, lysine, and methionine in follow-on (2) infant formula IF-A was significantly higher than in infant formula IF-B and IF-C (*p* < 0.05). In most cases, the content of essential amino acids was at the same level in the first and follow-on milk of selected producers. Significant differences were only noted in the case of a higher content of phenylalanine in follow-on milk from all three producers and tryptophan in infant formula IF-A, which was higher in the first-feeding milk (1). The results obtained on the content of essential amino acids in infant formulas do not differ significantly from the results obtained by other authors [25].

### 3.3. Non-Essential Amino Acid Profile

Table 2 shows non-essential amino acids in breast milk and infant formulas. The levels of alanine, glutamic acid, serine, and tyrosine (*p* < 0.05), as well as aspartic acid, glycine, and proline in human milk increase during the first three months of lactation. In their studies, Wu et al. [26] noted similar relationships in the case of a decrease in arginine and cysteine and an increase in aspartic acid, glutamic acid, and proline, whereas Wei et al. [27] noted a decrease in aspartic acid, serine, glutamic acid, glycine, alanine, tyrosine, and arginine. The differences in the changing values of individual non-essential amino acids could be reflected in the components of protein in breast milk at different stages of lactation. The predominant amino acid in breast milk is glutamic acid, which accounts for more than 50% of the total amount of amino acids. Glutamic acid is the most abundant amino acid in human milk and has important functions, including improving zinc absorption or stimulating nervous system function [27]. There was a significant increase in the level of glutamic acid depending on the lactation period. It is indicated that glutamic acid shows the highest increase in concentration and could rise almost 350% from 1 to 6 months of lactation [28,29].

In infant formulas, glutamic acid was the dominant amino acid, with relatively high levels of aspartic acid and proline. The amino acid composition of the infant formulas from the three manufacturers was similar. There was a higher content of aspartic acid in milk IF-A—both first (1) and follow-on (2)—than in milk IF-B and IF-C, as well as a higher content of glycine in milk IF-A for the first feeding (1) (*p* < 0.05). In addition, a higher content of selected amino acids was observed in follow-on (2) milk compared to the first feeding: proline (IF-A, IF-B, IF-C milk), serine (IF-A, IF-B milk), tyrosine (IF-A milk), and glutamic acid (IF-B, IF-C milk). Infant formulas contained higher amounts of alanine, cysteine, glutamic acid, and serine (*p* < 0.05) compared to breast milk. A similar trend was observed by Wei et al. [27].

Table 3 shows the mean values of the total amino acids (TAA), essential amino acids (EAA), non-essential amino acids (NEAA), and EAA to TAA ratios in the studied samples. The content of TAA in breast milk at different periods of lactation did not differ significantly (*p* < 0.05). On the other hand, changes in the qualitative composition of amino acids were noted. Colostrum had more essential amino acids, whereas mature milk was richer in non-essential amino acids. In addition, the total EAA to TAA ratio was higher in milk from the first lactation period. Lönnerdal et al. [9] reported a 7% lower EAA/TAA ratio in colostrum relative to the study conducted. The differences in the content of essential amino acids may be due to the variability of amino acid content during the circadian rhythm [24]. However, the authors obtained similar results for the EAA/TAA ratio in the 2nd and 3rd lactation periods. In contrast, the EAA/TAA ratio in the study by Feng et al. [20], in both transitional and mature milk, was at similar levels (50.17, 49.21%, respectively). Although the content of TAA, EAA and NEAA changes as breast milk matures, the EAA/TAA ratio was stable over time [9]

The ratio of EAA to TAA content was not significantly different in the infant formula from different manufacturers, both in first (1) and follow-on (2) milk. According to Codex Alimentarius recommendations, the EAA content of infant formula should be equal to or higher than that of breast milk [8,19]. It was reported that the EAA content of formula milk was the same or higher in infant formulas from different manufacturers. Ventura et al. [30], while studying the amino acid content of infant formulas, also took into account the degree of the hydrolysis of the powders. The authors obtained the most similar EAA/TAA ratio to the results of their own study in the case of infant formulas with a partial and complete degree of hydrolysis (46.72 and 55.02%, respectively).

### 3.4. Mineral Content

Table 4 shows the content of selected macrominerals (magnesium, Mg; calcium, Ca; sodium, Na; potassium, K) and microminerals (copper, Cu; manganese, Mn; iron, Fe; zinc, Zn) in breast milk and infant formulas.

It is assumed that the level of minerals decreases with the extension of lactation, which is primarily due to the continuous increase in the volume of milk by nursing mothers and the dilution of the concentration of minerals [27]. The concentration of Mg in breast milk is homeostatically regulated, ensuring the stability of this element regardless of metabolic or dietary factors [31]. The magnesium content in both colostrum and transitional/ mature milk was 3.41 and 3.02 mg/100 mL, respectively. According to the literature, the concentration of Mg in breast milk ranges from 1 to 5 mg/100 mL, but in most studies, the median concentration of this element is around 3 mg/100 mL [31,32], which is in line with the results of the research conducted. The literature reports that the concentration of Mg in breast milk is constant and independent of the lactation period [32], However, in the study conducted, colostrum contained more Mg than transitional and mature milk (*p* < 0.05). In the literature, there are slight upward or downward trends in Mg content, but the differences are not significant. The changes in composition relative to the lactation period can be explained by the interindividual variation of women participating in the study (age, BMI) [32]. The Mg content of infant formula was higher than that of breast milk (*p* < 0.05). In addition, there was a higher Mg content in the follow-on (2) milk of producers IF-A, IF-B (*p* < 0.05), and IF-C (*p* > 0.05). The increased amount of Mg in infant formulas is a result of less absorption of this element compared to breast milk.

The calcium content in colostrum was higher compared to transitional and mature milk (*p* < 0.05). The results of a meta-analysis by Rios-Leyvraz and Yao [33] report that the Ca content of colostrum is 26.9 mg/100 mL, whereas that of transitional and mature milk is about 26 mg/100 mL. These values are similar to the results obtained in our study. The concentration of total Ca increases during the first days of lactation (colostrum), and then a gradual decrease in its content is noted [32]. Calcium is the second dominant micromineral in breast milk. According to EFSA [34], an infant should consume 200 mg Ca per day to ensure normal skeletal development. This amount can be provided by the consumption of 800 mL of milk per day if it contains a Ca concentration of 250 mg/L. Breast milk—both colostrum and transitional/ mature milk—met this standard, which allows for an adequate supply of this element during breastfeeding. Compared with breast milk, infant formula contained twice as much Ca on average. EFSA [35] reports that the bioavailability of Ca from human milk is 58%, while that of infant formula is 38%. Therefore, infant formulas available on the market are fortified with a much higher Ca content to ensure the adequate intake of this element by infants. According to issued directives, powdered infant formulas should contain between 50 and 140 mg Ca/100 kcal of product [36], and the infant formulas tested met this standard.

There were differences in Na and K content relative to the lactation period; colostrum contained 68% more Na and 20% more K than transitional and mature milk (*p* < 0.05). A similar downward trend was observed by Kim and Yi [6]. In contrast, Fernandes Massmann et al. [37] indicated higher sodium and lower potassium concentrations in colostrum relative to transitional and mature milk. There is no conclusive information in the literature regarding the electrolyte content of breast milk, as their levels depend on a number of factors, including increased stress or mastitis. The WHO report states that the concentration of sodium in breast milk is in the range of 6–17 mg/100 mL, while the EFSA reports 14–16 mg/100 mL [31]. Values obtained by other authors significantly exceed these standards [38,39]. The sodium content of infant formulas did not differ from that of breast milk. Moreover, both first (1) and follow-on (2) milk contained the same Na values. By contrast, breast milk—both colostrum and transitional/ mature—contained less potassium relative to infant formulas (*p* < 0.05).

The Fe content of breast milk varied significantly in regard to the lactation period; it was 0.032 µg/100 mL in colostrum and 0.023 µg/100 mL in transitional and mature milk. Similar trends were reported by Kim and Yi [6]. The decrease in Fe concentration with the progress of lactation is mainly due to the decreasing content of protein in mature milk, to which most Fe is bound [27]. The Fe content in breast milk is at a very low level (about 0.04 ug/100 mL), but this is sufficient for infants due to its high bioavailability (20–50%) [6]. Due to the lower bioavailability of Fe in the infant formulas, each of the tested formulas (IF-A, IF-B, IF-C) contained significantly higher values of this element. Due to the lower absorption of Fe from infant formulas (4–7%), it is necessary to implement supplementation in infants fed infant formula after 4 months of age [31]. A similar trend was noted for Zn; colostrum contained twice as much of this element compared to transitional and mature milk—a finding also confirmed by Maharani et al. [40]. The same observation for Fe was reported for infant formulas, in which the concentration of Zn significantly exceeded that of breast milk (*p* < 0.05). This is also related to lower Zn absorption from infant formulas than from breast milk. In addition, the lactation period had an effect on Cu levels in breast milk. Colostrum contained 35% more Cu than transitional and mature milk, as confirmed by a study by Winiarska-Mieczan [41]. Higher Cu contents in infant formulas are associated with the lower bioavailability of this element compared to breast milk, as is the case with Fe and Zn.

In the study conducted, Mn content was higher in colostrum than in transitional and mature milk, which confirms the results of Mitchell et al. [42]. It is reported that Mn concentration is highest in colostrum, and in mature milk, it remains relatively stable and does not change over time [42]. There were high differences in Mn content between breast milk and infant formulas. Colostrum contained about two times less Mn than first-feeding (1) infant formulas, and transitional and mature milk contained about three times less, relative to follow-on infant formulas. Until now, it was considered that Mn was better absorbed from breast milk, but currently, there is no indication of large differences in the bioavailability of Mn in human milk and infant formulas [41]. Maruszewska [36] reports that the manufacturers’ suggested daily serving of first-feeding (1) infant formulas covers 50% of Mn requirements. It is important to follow producers’ recommendations for the preparation of infant formula milk, as infant exposure to high Mn content may be associated with adverse neurodevelopmental effects [7].

### 3.5. Limitations

This study has certain limitations. The research did not assess the diet of breastfeeding women. However, it is reported that protein and its amino acid composition are constant components in breast milk, and their content and variability are physiologically determined. Many researchers confirmed that the amino acid content of the diet did not directly relate to the amino acid profile in breast milk. Problems with protein content in milk can occur when mothers are malnourished; however, one of the inclusion criteria was the absence of disease. The content of minerals is also physiologically determined to a greater extent. There were some limitations related to collecting milk samples. Milk samples were not collected at regular times, because the women fed their babies on demand. The levels of some amino acids change in a circadian rhythm, and depending on the time of food intake, it can vary significantly. However, during the research, great attention was paid to the comfort of the mother, so no modifications were made to the method or frequency of feeding. Moreover, the collection of samples took place immediately after feeding; hence, only hindmilk with higher amounts of nutrients, including protein, was collected. For this reason, it was difficult to assess the total composition of the milk in terms of the studied components. This procedure was used in order not to interfere with the habitual feeding of infants and to provide them with sufficient milk.

## 4. Conclusions

Amino acids and minerals play an important role in the development of infants. Their levels in human milk are physiologically determined and change with the stage of lactation. In this study, both breast milk and infant formulas were rich sources of amino acids and minerals. More protein and essential amino acids were found in colostrum compared to transitional and mature milk. Moreover, infant formulas contained more amino acids; however, the EAA/TAA ratio was at the same level as in human milk. In relation to minerals, infant formulas contained several higher amounts of micro and macrominerals. Infant formulas were produced according to established standards, and their amino acid and mineral compositions were comparable. The follow-on IF-A infant formula contained more phenylalanine, proline, serine, and tyrosine. Breast milk, however, is most recommended for infant nutrition for the first 6 months of life due to its unique nutritional value. Infant formula is an alternative to breast milk when breastfeeding is not possible. Despite the different amino acid and mineral components in infant formulas, the levels cover children’s nutritional needs, ensuring proper development. Future studies are warranted to study the bioavailability of nutrients from human milk and infant formulas to fully understand their composition and absorption in a baby’s body.

## Figures and Tables

**Table 1:** The nutritional value of the first and follow-on infant formulas studied.

Nutritional Value in 100 g of Powder	First Feeding	Follow-on Feeding
Energy (kcal)	478–489	484–500
Fat (g), of which	23.7–27.7	24.2–24.5
Saturated fatty acid (g)	7.9–11.4	8.9–9.4
Monounsaturated fatty acids (g)	9.4–10.8	8.0–10.3
Polyunsaturated fatty acids (g)	4.3–5.0	4.3–5.3
Carbohydrates (g), of which	53.6–57.8	54.5–62
Sugars (g)	53.6–57.8	54.5–62
Protein (g)	7.3–10.5	9.4–9.6
Vitamin A (µg)	420–470	450–460
Vitamin D (µg)	6.7–11.6	11.0–12.5
Vitamin C (mg)	67–110	56–90
Riboflavin (mg)	0.9–1.2	1.1–1.2
Vitamin B12 (µg)	0.7–1.3	1.2–1.4
Calcium (mg)	330–442	460–490
Iron (mg)	3.8–5.2	4.6–6.0
Zinc (mg)	3.5–5.4	3.6–3.7
Iodine (µg)	94–130	105–107
Preparation of 100 mL of milk	12.9–13.8 g of powder + 90 mL of water	12.9–14.4 g of powder + 90 mL of water

**Table 2:** The content of essential and non-essential amino acids in human milk depending on the lactation period and in infant formulas depending on the type of the milk (first feeding—1 and follow-on feeding—2), g/100 g of protein.

Amino Acid Content		HM		Infant Formula					
				IF-A		IF-B		IF-C	
		I	II/III	1	2	1	2	1	2
His	Essential amino acids	2.50 ± 0.97 ^b^	2.50 ± 0.80 ^B^	3.35 ± 0.39 ^a^	3.43 ± 0.17 ^A^	2.74 ± 0.77 ^b^	3.12 ± 1.41 ^B^	2.64 ± 0.32 ^b^	2.89 ± 0.90 ^B^
Trp		1.58 ± 0.18 ^a^*	1.28 ± 0.41 ^B^	1.69 ± 0.08 ^a^*	1.52 ± 0.20 ^A^	1.45 ± 0.07 ^b^	1.34 ± 0.37 ^A^	1.49 ± 0.04 ^b^	1.60 ± 0.35 ^B^
Leu		8.31 ± 1.66 ^d^	7.34 ± 2.37 ^A^*	8.54 ± 1.89 ^a^	8.47 ± 0.96 ^A^	8.24 ± 3.10 ^b^	8.36 ± 1.29 ^A^	7.96 ± 2.05 ^c^	8.25 ± 2.81 ^A^
Ile		6.15 ± 1.41 ^a^	5.17 ± 1.02 ^A^*	5.25 ± 0.99 ^a^	5.14 ± 0.80 ^B^	5.09 ± 2.14 ^a^	5.38 ± 1.22 ^A^	5.15 ± 0.65 ^a^	5.33 ± 1.15 ^B^
Phe		5.00 ± 0.65 ^a^	3.85 ± 1.16 ^A^*	4.65 ± 0.31 ^b^	5.00 ± 0.61 ^A^*	4.37 ± 0.76 ^b^	4.89 ± 0.85 ^A^*	4.33 ± 0.79 ^b^	4.93 ± 1.46 ^A^*
Thr		4.42 ± 1.42 ^b^	3.95 ± 1.09 ^B^*	5.29 ± 0.67 ^a^	5.19 ± 0.84 ^A^	5.16 ± 1.74 ^a^	5.01 ± 0.44 ^A^	5.06 ± 0.98 ^a^	5.13 ± 1.33 ^A^
Lys		5.36 ± 1.41 ^b^	5.33 ± 1.23 ^C^	7.88 ± 2.56 ^a^	7.96 ± 0.56 ^A^	6.99 ± 1.77 ^a^	7.23 ± 1.23 ^B^	6.92 ± 2.04 ^a^	7.17 ± 1.70 ^B^
Val		5.47 ± 1.08 ^b^	4.60 ± 1.20 ^A^*	5.31 ± 1.10 ^a^	5.52 ± 1.16 ^A^	5.18 ± 1.02 ^a^	5.59 ± 0.86 ^A^	5.21 ± 1.75 ^a^	5.42 ± 0.88 ^A^
Met		2.14 ± 0.72 ^b^	1.57 ± 0.69 ^B^*	2.54 ± 0.31 ^a^	2.85 ± 0.88 ^A^	2.63 ± 0.89 ^a^	2.57 ± 0.22 ^B^	2.43 ± 0.54 ^a^	2.50 ± 0.25 ^B^
Ala	Non-essential amino acids	3.50 ± 0.99 ^a^	4.30 ± 1.34 ^A^*	3.95 ± 0.10 ^a^	3.77 ± 0.77 ^B^	3.73 ± 1.08 ^a^	3.67 ± 0.91 ^B^	3.72 ± 0.80 ^a^	3.75 ± 0.61 ^B^
Arg		3.07 ± 0.70 ^b^	2.89 ± 1.15 ^B^	4.57 ± 0.31 ^a^	3.77 ± 0.28 ^A^	2.99 ± 0.78 ^b^	3.46 ± 0.37 ^A^	2.96 ± 0.56 ^b^	3.23 ± 0.45 ^A^
Asp		6.56 ± 1.94 ^c^	6.62 ± 1.65 ^C^	8.43 ± 1.37 ^a^	8.24 ± 1.21 ^A^	7.60 ± 2.57 ^b^	7.63 ± 1.35 ^B^	7.60 ± 1.03 ^b^	7.73 ± 1.08 ^B^
Cys		3.45 ± 0.95 ^a^	2.95 ± 0.85 ^A^	2.42 ± 0.47 ^b^	2.39 ± 0.38 ^B^	2.31 ± 0.92 ^b^	2.20 ± 0.18 ^B^	2.24 ± 0.80 ^b^	1.96 ± 0.77 ^C^
Glu		11.12 ± 3.09 ^b^	12.14 ± 3.23 ^B^*	13.19 ± 2.18 ^a^	14.05 ± 2.14 ^A^	12.77 ± 2.47 ^a^	13.73 ± 1.94 ^A^*	12.74 ± 2.00 ^a^	13.37 ± 2.50 ^B^
Gly		2.37 ± 0.47 ^a^	2.68 ± 0.95 ^A^	2.14 ± 0.18 ^a^	2.20 ± 0.03 ^A^	2.02 ± 0.75 ^b^	2.18 ± 0.56 ^A^	2.04 ± 0.04 ^b^	2.12 ± 0.75 ^A^
Pro		7.48 ± 2.40 ^a^	8.10 ± 2.72 ^A^	7.34 ± 1.03 ^a^	8.66 ± 1.26 ^A^*	7.35 ± 1.51 ^a^	8.53 ± 1.31 ^A^*	7.19 ± 2.04 ^a^	7.85 ± 2.03 ^A^
Ser		3.97 ± 2.01 ^b^	4.25 ± 1.37 ^B^*	4.73 ± 0.91 ^a^	5.04 ± 0.61 ^A^*	4.75 ± 0.85 ^a^	4.98 ± 0.16 ^A^*	4.61 ± 1.37 ^a^	4.81 ± 1.06 ^B^
Tyr		6.60 ± 1.75 ^a^	3.71 ± 0.85 ^A^*	3.85 ± 0.82 ^a^	4.32 ± 0.48 ^B^*	3.89 ± 0.04 ^a^	4.39 ± 1.00 ^B^*	3.87 ± 0.84 ^a^	4.03 ± 0.95 ^B^

Abbreviation: HM-I—human milk (1st stage of lactation), HM-II/III—human milk (2nd and 3rd stage of lactation); IF-A-1/IF-B-1/IF-C-1—infant formulas from different manufacturers for the first feeding; IF-A-2/IF-B-2/IF-C-2—infant formulas from different manufacturers for the follow-on feeding. Means with different letters (a,b,c/A,B,C) are significantly different (*p* < 0.05). Means a,b,c specify differences between HM-I, IF-A-1, IF-B-1, and IF-C-1; means A,B,C specify differences between HM-II/III, IF-A-2, IF-B-2, and IF-C-2. Values marked with * indicate differences in amino acid content between breast milk from the I and II/III lactation period or between first and follow-on infant formulas.

**Table 3:** The mean values of the total (T), essential (E), non-essential (NE), and essential to total amino acid (AA) ratio in human milk and infant formulas.

Amino Acid Content		HM		Infant Formula					
				A		B		C	
		I	II/III	1	2	1	2	1	2
TAA	g/100 g of protein	86.47 ^c^	85.81 ^C^	95.12 ^a^	97.52 ^A^	89.19 ^b^	94.20 ^A^*	88.23 ^b^	92.13 ^A^
EAA		40.93 ^b^*	35.59 ^B^	44.50 ^a^	45.08 ^A^	41.78 ^b^	43.43 ^A^	41.26 ^b^	43.28 ^A^
NEAA		45.54 ^b^	50.22 ^B^*	50.62 ^a^	52.44 ^A^	47.41 ^b^	50.77 ^B^	46.97 ^b^	48.85 ^B^
EAA/TAA	%	47 ^a^	41 ^B^*	47 ^a^	46 ^A^	47 ^a^	46 ^A^	47 ^a^	47 ^A^

Abbreviation: HM-I—human milk (1st stage of lactation), HM-II/III—human milk (2nd and 3rd stage of lactation); IF-A-1/IF-B-1/IF-C-1—infant formulas from different manufacturers for the first feeding; IF-A-2/IF-B-2/IF-C-2—infant formulas from different manufacturers for the follow-on feeding, TAA—total amino acids, EAA—essential amino acids, NEAA—non-essential amino acids. Means with different letters (a,b,c/A,B,C) are significantly different (*p* < 0.05). Means a,b,c specify differences between HM-I, IF-A-1, IF-B-1, and IF-C-1; means A,B,C specify differences between HM-II/III, IF-A-2, IF-B-2, and IF-C-2. Values marked with * indicate differences in TAA, EAA, NEAA, and EAA/TAA content between breast milk from the I and II/III lactation period or between first and follow-on infant formulas.

**Table 4:** The content of selected macrominerals (Mg, Ca, Na, K) (mg/100 mL) and microminerals (Cu, Mn, Fe, Zn) (µg/100 mL) in human milk depending on the lactation period and in infant formulas depending on the type of the milk (first feeding—1 and follow-on feeding—1).

Sample		Human Milk		Infant Formula					
				A		B		C	
Type of Milk		I	II/III	1	2	1	2	1	2
Macrominerals, mg/100 mL	Mg	3.41 ± 0.65 ^b^*	3.02 ± 0.54 ^C^	6.25 ± 0.39 ^a^	7.64 ± 0.47 ^A^*	5.78 ± 0.75 ^a^	7.77 ± 1.04 ^A^*	6.02 ± 0.84 ^a^	6.70 ± 0.03 ^B^
	Ca	27.13 ± 5.07 ^b^	25.89 ± 4.13 ^C^	59.43 ± 1.14 ^a^	73.46 ± 7.83 ^B^*	60.77 ± 6.66 ^a^	88.22 ± 4.28 ^A^*	62.04 ± 6.00 ^a^	69.47 ± 2.15 ^B^
	Na	23.66 ± 7.19 ^a^*	14.07 ± 7.55 ^A^	17.32 ± 0.86 ^a^	21.41 ± 2.57 ^A^	19.62 ± 3.56 ^a^	24.93 ± 5.74 ^A^	16.12 ± 3.08 ^a^	18.45 ± 0.48 ^A^
	K	59.87 ± 11.60 ^b^*	49.84 ± 6.77 ^B^	71.51 ± 2.93 ^a^	88.12 ± 5.77 ^A^	77.93 ± 7.10 ^a^	92.01 ± 11.10 ^A^	69.30 ± 4.47 ^a^	85.43 ± 2.15 ^A^
Microminerals, µg/100 mL	Cu	0.04 ± 0.00 ^a^*	0.03 ± 0.00 ^A^	0.03 ± 0.00 ^a^	0.04 ± 0.01 ^A^	0.03 ± 0.01 ^a^	0.03 ± 0.00 ^A^	0.05 ± 0.01 ^a^	0.04 ± 0.02 ^A^
	Mn	0.31 ± 0.07 ^c^	0.20 ± 0.09 ^B^*	0.69 ± 0.07 ^b^	0.72 ± 0.05 ^A^	0.61 ± 0.04 ^b^	0.67 ± 0.07 ^A^	0.73 ± 0.09 ^a^	0.69 ± 0.03 ^A^
	Fe	0.03 ± 0.00 ^b^	0.02 ± 0.00 ^B^*	0.08 ± 0.01 ^a^	0.11 ± 0.01 ^A^*	0.07 ± 0.02 ^a^	0.09 ± 0.02 ^A^*	0.07 ± 0.02 ^a^	0.09 ± 0.01 ^A^*
	Zn	0.33 ± 0.11 ^b^*	0.15 ± 0.07 ^C^	0.62 ± 0.10 ^a^	0.74 ± 0.07 ^A^*	0.49 ± 0.06 ^b^	0.56 ± 0.05 ^B^	0.59 ± 0.10 ^a^	0.76 ± 0.01 ^A^*

Abbreviation: HM-I—human milk (1st stage of lactation), HM-II/III—human milk (2nd and 3rd stage of lactation); IF-A-1/IF-B-1/IF-C-1—infant formulas from different manufacturers for first feeding; IF-A-2/IF-B-2/IF-C-2—infant formulas from different manufacturers for follow-on feeding. Means with different letters (a,b,c/A,B,C) are significantly different (*p* < 0.05). Means a,b,c specify differences between HM-I, IF-A-1, IF-B-1, and IF-C-1; means A,B,C specify differences between HM-II/III, IF-A-2, IF-B-2, and IF-C-2. Values marked with * indicate differences in minerals content between breast milk from the I and II/III lactation period or between first and follow-on infant formulas.

## Data Availability

The data presented in this study are available on reasonable request from the corresponding author.
